# Assessing the Impact of School-Based Greenness on Mental Health Among Adolescent Students in Ontario, Canada

**DOI:** 10.3390/ijerph16224364

**Published:** 2019-11-08

**Authors:** Sebastian A. Srugo, Margaret de Groh, Ying Jiang, Howard I. Morrison, Hayley A. Hamilton, Paul J. Villeneuve

**Affiliations:** 1 Centre for Surveillance and Applied Research, Public Health Agency of Canada, Ottawa, ON K1A 0K9, Canada; sebastiansrugo@gmail.com (S.A.S.); margaret.degroh@canada.ca (M.d.G.); ying.jiang@canada.ca (Y.J.); howard.morrison@canada.ca (H.I.M.); 2 Institute for Mental Health Policy Research, Centre for Addiction and Mental Health, Toronto, ON M5S 2S1, Canada; hayley.hamilton@camh.ca; 3 Dalla Lana School of Public Health, University of Toronto, Toronto, ON M5T 3M7, Canada; 4 School of Mathematics and Statistics, Carleton University, Ottawa, ON K1S 5B6, Canada

**Keywords:** built environment, schools, mental health, adolescent, survey

## Abstract

Neighbourhood greenness has been frequently associated with improved mental health in adulthood, yet its impact among youth is less clear. Additionally, though youth spend large portions of time at school, no study has investigated associations between school-based measures of greenness and students’ mental health in Canada. We addressed this gap by linking participant responses from the 2016–2017 Ontario Student Drug Use and Health Survey to school-based features of the built environment. Our analyses included 6313 students, ages 11–20. Measures of greenness were the mean and max of the annual mean Normalized Difference Vegetation Index within 500 m and 1000 m from the centroid of the school postal code. Measures of mental health included: serious psychological distress (Kessler 6-item Psychological Distress Scale), self-rated mental health (using a five-point Likert scale), suicide ideation, and suicide attempt. In our study population, the prevalence of serious psychological distress and low self-rated mental health was 16.7% and 20.3%, respectively. Suicide ideation was reported by 13.5% of participants, while 3.7% reported a suicide attempt. Quantity of greenness was similar between schools in the lower and upper quartiles. In logistic regressions, we found no association between objective school-based greenness and mental health, as assessed by multiple measures, both before and after adjustment. Null findings held true after stratification by season, as well. Whether other characteristics of school greenness (such as type, quality, or access and use) are more impactful to students’ mental health should be a focus of future analyses.

## 1. Introduction

The transition between childhood and adulthood is a period of life characterized by rapid physical, psychological, and cognitive development, and lays a foundation for future mental health and resilience [1,2]. An estimated 70% of mental health problems first appear during this developmental period [3]. According to a recent provincial survey, approximately 1 in 3 Ontario middle- and high-school students (grades 7–12) indicated moderate to serious psychological distress, with prevalence increasing greatly by grade and over time [4]. Mental illness is responsible for the most years of life lost due to disability and premature mortality of any other condition among Canadian youth aged 10–24 [5]. Nevertheless, it is still unclear which factors contribute most to mental illness in this age group and to what extent it can be prevented.

Built environments have been shown to have an important influence on mental health. Recent studies have found that quantity and access to neighbourhood green spaces are associated with a reduced risk of stress, psychiatric morbidity, psychological distress, depression, and anxiety among adults [6,7,8]; this relationship has been posited to be a result of increased physical activity, social cohesion, and direct psychological benefits of natural outdoor environments [6]. Literature on the relationship between greenness and mental health among youth suggests that greenness has a beneficial role [9], though the few studies that have been conducted in Canada are inconsistent [10,11]. One found a negative association between urban/suburban residential greenness and health-related quality of life among youth (aged 8–14) in the province of Ontario [10], while the other found no association between residential greenness and mental health among young and older adults (aged 18–77) in Ottawa, Ontario [11]. Additionally, middle- and high-school students spend large amounts of time at and around schools, yet only three studies have examined the impact of school-based greenness on students’ mental health. Akpinar (2016) found subjective school greenness to only be associated with perceived restorativeness of green spaces and not health nor mental health among 223 students in Turkey [12]. Li and Sullivan (2016) found window views of green landscapes promoted attention restoration based on a digit memory test and recovery from stress based on physiological markers (skin conductance, body temperature, and heart rate variability) among 94 students from five high schools in central Illinois [13]. Lastly, Ribeiro and colleagues (2019) found modest inverse associations between having green spaces (binary) at 400 m or 800 m around elementary schools and proxies of chronic stress (allostatic load) based on biological markers among 3108 7-year-old students in Portugal [14]. While these studies have provided valuable insights, they have been limited by small sample sizes, their measures of greenness (one of which is subjective and the other binary), and/or their measures of mental health (two of which used physiological markers which, while objective, fail to account for the participant’s feelings about their own mental health and may be biased by variability throughout the day and increased stress from study participation). Furthermore, none of these studies were undertaken in Canada, where the association may differ due to its unique climate and more seasonal greenery.

In our study, we used cross-sectional data collected from the 2016–2017 Ontario Student Drug Use and Health Survey (OSDUHS) that were linked with school-based built environment data from the Canadian Urban Environmental Health Research Consortium data repository. These data allowed us to investigate whether objective school-based greenness is associated with indicators of mental health (such as serious psychological distress, self-rated mental health, and suicide ideation and attempt) among Ontario middle- and high-school students aged 11–20. 

## 2. Materials and Methods 

### 2.1. Data Sources

The OSDUHS was first administered in 1977 and is the longest-running school survey of adolescents in Canada. Detailed description of the methods used in the survey can be found elsewhere [4]. Briefly, the OSDUHS is a biennial cross-sectional survey of Ontario students enrolled in grades 7–12 (11–20 years old) and is completed anonymously. The survey employs a stratified (region and school-level), two-stage (school and class) cluster sample design, devised to produce a representative sample of the approximately 1 million students in grades 7–12 in publicly-funded schools in Ontario. For our study, we used student data from the 2016–2017 cycle of the OSDUHS to take advantage of recently-constructed built environment exposure surfaces. In this cycle, 11,435 students drawn from 764 classes in 214 schools participated in the self-administered, anonymous survey between November 2016 and June 2017. This represented a response rate of 61%, 94%, and 61% for students, classes, and schools, respectively. To include as many topics as possible within the confines of a fixed-class period and while reducing time-burden, two questionnaire forms differing in half of the items were randomized among students; only one of the two included information on mental health, therefore our analysis was restricted to this half-sample of adolescents (*N* = 6313). Built environment data from Ontario in 2016 (such as greenness and neighbourhood-level socio-economic status) were obtained from the Canadian Urban Environmental Health Research Consortium (CANUE).

The Research Ethics Boards at the Centre for Addiction and Mental Health and York University, as well as Research Review Committees at 31 school boards, approved the 2016–2017 OSDUHS protocol. This specific study was approved by Carleton University’s Research Ethics Board (CUREB).

### 2.2. Measures of Mental Health (Outcome)

#### 2.2.1. Psychological Distress

The Kessler 6-item Psychological Distress Scale (K6) was employed as a validated measure of non-specific psychological distress in the past month, including symptoms of anxiety and depression [4,15]. The K6 has previously been shown to be a valid assessment tool of psychological wellbeing among adolescents [16,17], and showed high internal reliability in our study sample (Cronbach’s α = 0.88). Each item was assessed on a five-point Likert scale, where responses were scored from 0–4 and summed together to a range of 0–24 for students who completed all six items; serious distress was classified as a total score of 13 or more, as suggested by Kessler and colleagues [15]. 

#### 2.2.2. Self-Rated Mental Health

On a five-point Likert scale, participants were asked to rate their mental or emotional health. Responses of fair and poor were categorized as low self-rated mental health, while those of good, very good, and excellent were categorized as high. This measure of general mental health has previously been validated in Canada [18].

#### 2.2.3. Suicide Ideation or Attempt

Suicide ideation or attempt were based on ‘*yes*’ or ‘*no*’ responses to: “*In the last 12 months, did you seriously consider attempting suicide?*” and “*In the last 12 months, did you actually attempt suicide?*”, respectively. Similar wording has been used elsewhere [19,20]. 

### 2.3. School-Based Greenness (Exposure)

School-based greenness was assessed based on the mean or maximum of the annual mean Normalized Difference Vegetation Index (NDVI) within 500 m or 1000 m from the centroid of the six-character school postal code (representing green space within a 10-min or 20-min walk, respectively). Many urban and suburban schools in Ontario have their own postal codes, whereas rural schools are likely to be included in the postal code of the surrounding area. Nevertheless, due to being weighted to the spatial distribution of populations within the postal code boundary [21], the centroid is an accurate representation of the location of residents within the area and should thus approximate the school location, both in urban and rural areas. As NDVI is a widely-used measure of greenness, we employed this measure to be able to compare our results with similar studies in different settings and age-ranges; the index employs the difference in radiation between red and near-infrared wavelengths—used by photosynthetic plants to absorb and emit radiation, respectively—to identify green vegetation using satellite data [22]. The measure of greenness ranges from −1 (no greenness) to 1 (all green) and was categorized into quartiles. NDVI data from 2016 were taken from the United States Geological Survey Landsat 8 satellite with a spatial resolution of 30 m and accessed via Google Earth Engine [23,24,25,26]. NDVI data from the Moderate Resolution Imaging Spectroradiometer (MODIS) onboard the TERRA satellites and accessed via Google Earth Engine [23,27] were also used in a sensitivity analysis. More information about the greenness measures can be found elsewhere [28]. Season, determined by date of questionnaire completion, was classified as fall/winter (November–February) or spring/summer (March–June). No questionnaires were completed between July and October.

### 2.4. Covariates

#### 2.4.1. Socio-Economic Status

The Material and Social Deprivation Index (MDSI), a proxy for neighbourhood-level socio-economic status (SES) which included information on six socio-economic indicators (such as neighbourhood income, education, proportion of single-parent families, etc.), was linked to the dataset [24,29,30]. The material and social deprivation dimensions were each categorized into tertiles (from low (T1) to high (T3) deprivation) and combined into one measure with three categories: low, medium, and high material and social deprivation, similar to methods previously published [31]. The OSDUHS questionnaire also included self-reported socio-economic status (10-point scale based on how ‘well off’ the participant believed their family to be). We grouped the scale by twos to create a five-point scale, since the five-point scale was significantly associated with both our main outcome (serious psychological distress) and some measures of greenness, while the 10-point version was not. 

#### 2.4.2. Walkability

The walkability of school neighbourhoods in 2016 was compiled by researchers through the Canadian Active Living Environments (Can-ALE) dataset and made available on the CANUE data repository [32,33]. The continuous measure of degree of walkability was calculated based on the summed z-scores of points of interest, intersection density, dwelling density, and transit around the neighbourhood centroid [34]. A z-score greater than zero would represent a neighbourhood that is more walkable than the Canadian average.

#### 2.4.3. Demographics and Other Covariates

Additional covariates selected from the OSDUHS survey were age (categorized as: 11–12, 13–14, 15–16, and ≥17 years); sex; region (Greater Toronto Area, including Toronto, Durham Region, York Region, Peel Region, and Halton Region; Northern Ontario, including Parry Sound District, Nipissing District, and farther north; Western Ontario, including Dufferin County and farther west; and Eastern Ontario, including Simcoe County and farther east); home language (English and/or French only, as well as Other); years since immigration to Canada (Canadian-born, ≤2 years, 3–5 years, 6–10 years, and ≥11 years); physical activity; outdoor play; frequency of being bullied, including traditional and cyberbullying; and ethno-racial background (White, Asian, Black, Indigenous, Latin American, and multiple). Physical activity (PA) was assessed with the question: “*On how many of the last 7 days were you physically active for a total of at least 60 min each day?*”. Responses equal to seven days were coded as ‘meeting guidelines’ based on Canadian PA guidelines [35]; all other responses were coded as ‘not meeting guidelines’. Outdoor play was defined as being physically active outside after school at least one day over the last five school days, excluding travel time from school. For ethno-racial background, participants could report more than one category to which they identified; if more than one was reported (even if the second category was ‘not sure’), the participant was placed in the ‘multiple’ ethno-racial background category. However, if the respondent identified as Indigenous, they were placed in this category regardless of other ethno-racial backgrounds reported due to the unique history and culture of this group in Canada. 

### 2.5. Statistical Analyses

All analyses were conducted using SAS Enterprise Guide 5.1 (SAS Institute, Cary, NC, USA). Descriptive statistics were calculated and presented with the distribution of mean greenness at 500 m across these categories. Logistic regressions, which applied the generalized estimating equations (GEE) method with an independent covariance structure to account for the clustering of students in schools, were employed to examine the adjusted odds of having serious psychological distress, a proxy for mental health, among students by objective school-based greenness (defined by four NDVI measures in separate models). Models were adjusted for age, sex, ethno-racial background, self-reported SES, and season. We adjusted for these variables based on their associations with both the exposure and outcome (i.e., due to confounding), except in the case of sex, which adjusted for a large imbalance in the outcome between males and females. Multiple sensitivity analyses were conducted; models were re-run with different measures of mental health (self-rated mental health, suicide ideation, or suicide attempt), different greenness data (MODIS satellite), different combinations of adjustment factors (including home language (as a proxy for minority status), years in Canada, bullying (including traditional and cyber), and walkability) and interaction terms (between greenness and: SES (self-reported SES or MDSI tertiles), physical activity, outdoor play, region, or season), and stratified by season to determine whether these changes affected overall conclusions. 

## 3. Results

**Figure 1:**
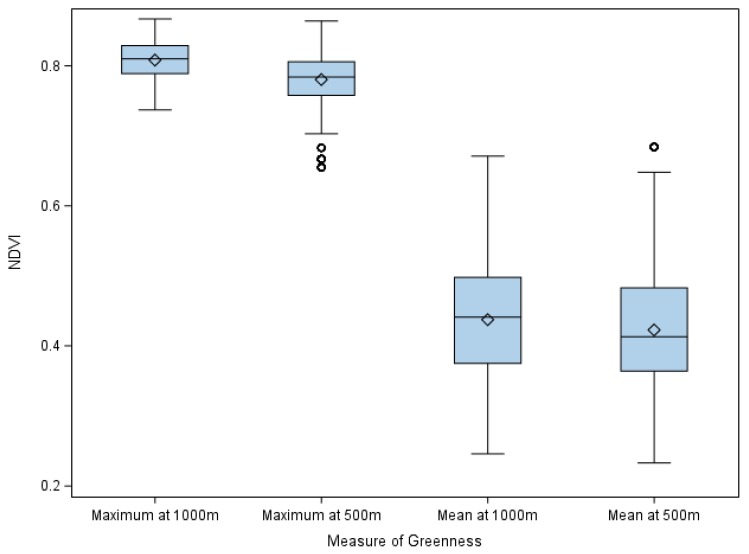
Distributions of school-based greenness by measure. Median (interquartile range) for maximum NDVI at 1000 m and 500 m and mean NDVI at 1000 m and 500 m are: 0.81 (0.04), 0.78 (0.05), 0.44 (0.12), and 0.41 (0.12), respectively. Abbreviations: NDVI, Normalized Difference Vegetation Index.

Sample characteristics are presented in Table 1. The majority of participants were 15 years old or older, White, or female. Most students did not meet the Canadian physical activity guidelines but played outside at least once in the previous five school days. Most spoke English and/or French exclusively at home, were Canadian-born, and self-reported high SES. Approximately 16.7% and 20.3% of students reported serious psychological distress and low self-rated mental health, respectively. Suicide ideation was indicated by 13.5% of participants, while 3.7% reported a suicide attempt. School-based greenness was similar between the lower and upper quartiles of each measure (average interquartile range of 0.08 in a scale ranging from −1 to 1; Figure 1).

Data from logistic regressions are shown in Table 2. Odds of serious psychological stress increased with age and were significantly higher for females compared to males (data not shown). When looking at ethno-racial background, only Indigenous students reported more serious psychological stress compared to White students (data not shown). A very strong relationship was observed according to self-reported socio-economic status (SES), with those in the lowest category almost 10 times more likely to report serious psychological stress compared to those in the highest category (data not shown). None of the models showed an association between any greenness measure and serious psychological distress. Sensitivity analyses replicated our null findings when the models were re-run with different measures of mental health (Table 2), different greenness data, and different combinations of adjustment factors and interaction terms (data not shown). Furthermore, stratification by season produced similar null findings. In a post hoc sensitivity analysis, employing the 10-point SES scale—instead of the five-point version—did not change the results of the logistic regressions. In a separate post hoc sensitivity analysis, we refit the models using the workable exchange covariance matrix and found that the standard errors (and associated confidence intervals) were essentially unchanged. Due to the nature of the data, and students being provided the surveys at the same time, we deemed the autoregressive covariance matrix to be unsuitable. We further considered multi-level modelling, but again, this analytic approach did not fundamentally change our findings.

## 4. Discussion

In this study, we examined the relationship between school-based greenness and mental health in a large sample of middle- and high-school students in publicly-funded schools in Ontario. To our knowledge, this is the first study to assess the impact of objective greenness around the school neighbourhood on mental health in Canada. We found no association between objective quantity of greenness around the school neighbourhood and students’ mental health, as assessed by multiple measures, both before and after adjustment. 

Several potential theories may explain why the school greenness-mental health relationship was null in our study. First, not all greenness is equal. A study of approximately 5200 Washington State residents found no relationship between overall residential-neighbourhood greenness (combining urban greenspaces, forests, rangelands, agricultural lands, and wetlands) and measures of mental or general health; however, when stratified by greenspace type, significant positive associations emerged between forests, urban greenspaces, and mental health [36]. Similarly, a study employing data from 3103 counties in the United States found that forests and shrubs were negatively associated with health-care spending, though grass-land cover had no association to the outcome [37]. Further, in an Australian study, quality, not quantity, of greenspaces was associated with low psychological distress, regardless of use [38]. These data suggest that the type and quality of greenness are important factors that modulate the impact of greenness on mental health. Our study was limited by employing a measure of overall greenness alone, and one that was objective, rather than subjective, which could not address subjective quality [6]. 

Second, we found relatively little variation in greenness between the schools in the lower and upper quartiles. Since most schools are similarly ‘green’, our ability to identify a significant association may have been limited.

Third, greenspace access and use may be important mediators of the association between greenness and health. While adolescents spend a large portion of time in school, most school hours are spent indoors and not out in the school neighbourhood. Although the presence of greenness alone can improve mental health through perceived restorative quality and reduction of air and noise pollution, greenspace use may also increase social cohesion and physical activity [39]. However, our study could not assess access nor use of greenspace among students, potentially masking any association. Still, it is presently unclear whether the presence of greenspace or its access and use is more impactful on mental health outcomes [40].

Additionally, there are other limitations to our study. We employed the centroid of the school postal code instead of the schoolyard boundary due to not having access to the shape of every schoolyard in the study. Therefore, smaller buffers may include part of the schoolyard, whereas the larger buffers are more likely to include the entire schoolyard. We found little difference in the results between the larger and smaller buffers, suggesting this is not a large source of bias. Further, we did not delve into how green design may be applied to school environments to increase mental health among students. These limitations, as well as the others outlined above, should be taken into consideration in future studies.

Lastly, notwithstanding the limitations of our study described above, school greenness may simply not be an important influence on students’ mental health. Exposure to school-based greenness is limited in Ontario by the seasons during the school year; students are exposed to little greenness during the late fall and winter, and they spend summers away from school, during which time greenness and greenspace use are presumably at their peak. Furthermore, the majority of a student’s week is spent at home, where residential greenness may be a more salient risk factor for mental health. Finally, other factors may be more impactful to mental health during adolescence, such as biological factors, social disadvantage, family conflicts, and bullying [41], leaving a smaller role for greenness in this relationship. 

## 5. Conclusions

Our study is the first to assess the relationship between objective school-based measures of greenness and students’ mental health in Canada. Though we found no association between these factors, it is still far from clear whether school greenness can impact students’ mental health due to the lack of research in this area. Future studies should elucidate this relationship, while focussing on overcoming the key limitations of our study in order to improve our understanding of this issue.

## Figures and Tables

**Table 1:** Sample characteristics of middle- and high-school students in Ontario who participated in the 2016 OSDUHS (*N* = 6313) and distribution of mean greenness (NDVI) at 500 m from 2016 CANUE data.

		Mean NDVI (500 m)		
	N (%)	25th Percentile	Median	75th Percentile
**Age (years)**				
11–12	804 (12.7)	0.38	0.43	0.48
13–14	2235 (35.4)	0.37	0.42	0.49
15–16	2100 (33.3)	0.35	0.41	0.49
≥17	1173 (18.6)	0.35	0.41	0.47
**Sex**				
Male	2730 (43.2)	0.36	0.42	0.48
Female	3583 (56.8)	0.36	0.41	0.48
**Ethno-racial background**				
White	3624 (59.7)	0.38	0.43	0.50
Asian	1134 (18.7)	0.34	0.39	0.44
Black	459 (7.6)	0.35	0.39	0.43
Indigenous ^a^	257 (4.2)	0.37	0.43	0.49
Latin American	114 (1.9)	0.35	0.41	0.43
Multiple ^b^	482 (7.9)	0.37	0.41	0.46
**Physical activity ^c^**				
Meeting guidelines	671 (10.7)	0.36	0.42	0.48
Not meeting guidelines	5575 (89.3)	0.36	0.41	0.48
**Outdoor play ^d^**				
At least once last school week	4700 (75.0)	0.36	0.42	0.49
None	1571 (25.1)	0.36	0.41	0.48
**Home language**				
English and/or French only	4732 (75.2)	0.37	0.42	0.50
Other	1561 (24.8)	0.35	0.40	0.45
**Years since immigration to Canada**				
Canadian-born	5227 (82.9)	0.37	0.42	0.49
≤2	148 (2.4)	0.36	0.39	0.43
3–5	197 (3.1)	0.35	0.41	0.47
6–10	367 (5.8)	0.35	0.41	0.46
≥11	368 (5.8)	0.35	0.41	0.46
**Self-reported SES ^e^**				
Lowest	40 (0.7)	0.35	0.41	0.47
Low	374 (6.1)	0.37	0.42	0.49
Middle	1587 (25.7)	0.36	0.41	0.49
High	3131 (50.8)	0.36	0.42	0.48
Highest	1034 (16.8)	0.36	0.41	0.48
**Material and Social Deprivation Index ^f^**				
Low deprivation	2047 (32.4)	0.38	0.44	0.50
Medium deprivation	2229 (35.3)	0.36	0.41	0.50
High deprivation	2037 (32.3)	0.34	0.40	0.44
**Region ^g^**				
Greater Toronto Area	2636 (41.8)	0.33	0.38	0.46
Northern Ontario	904 (14.3)	0.30	0.36	0.43
Western Ontario	1006 (15.9)	0.41	0.45	0.52
Eastern Ontario	1767 (28.0)	0.41	0.45	0.51
**Season of participation**				
Fall/winter (Nov–Feb)	3259 (51.6)	0.38	0.43	0.50
Spring/summer (Mar–Jun)	3054 (48.4)	0.33	0.39	0.46
**Mental health measures**				
Serious psychological distress ^h^	1018 (16.7)	0.36	0.41	0.48
Low self-rated mental health ^i^	1268 (20.3)	0.36	0.41	0.49
Suicide ideation	790 (13.5)	0.36	0.41	0.48
Suicide attempt	219 (3.7)	0.36	0.41	0.48

Abbreviations: OSDUHS, Ontario Student Drug Use and Health Survey; CANUE, Canadian Urban Environmental Health Research Consortium; NDVI, Normalized Difference Vegetation Index; SES, Socio-economic status. ^a^ Participants who identified themselves as Indigenous were classified as such, regardless of additional ethno-racial backgrounds selected. ^b^ Includes those who reported a specific ethno-racial background and ‘not sure’. ^c^ Meeting Canadian guidelines was based on being physically active for 60 min or more every day. ^d^ Outdoor play was defined as being physically active outside after school at least one day over the last five school days, excluding travel time from school. ^e^ Participants reported SES based on a 10-point scale; these categories were grouped by twos, creating the five-point scale shown. ^f^ The material and social deprivation dimensions were each categorized into tertiles (from low (T1) to high (T3) deprivation) and combined into one measure with three categories: low, medium, and high material and social deprivation. ^g^ Greater Toronto Area includes: Toronto, Durham Region, York Region, Peel Region, and Halton Region; Northern Ontario includes: Parry Sound District, Nipissing District, and farther north; Western Ontario includes: Dufferin County and farther west; and Eastern Ontario includes: Simcoe County and farther east. ^h^ Serious psychological distress is based on a score ≥13 on the Kessler 6-item Psychological Distress Scale (K6). ^i^ Low self-rated mental health based on responses of fair and poor on a five-point scale of mental or emotional health.

**Table 2:** Adjusted ^a^ odds of having poor mental health among middle- and high-school students in school neighborhoods by greenness quartile.

		Serious Psychological Distress		Low Self-Rated Mental Health		Suicide Ideation		Suicide Attempt	
Greenness Measures	Quartile Range	Cases (*N* = 5732)	aOR (95% CI)	Cases (*N* = 5881)	aOR (95% CI)	Cases (*N* = 5521)	aOR (95% CI)	Cases (*N* = 5535)	aOR (95% CI)
Max NDVI (1000 m)	0.74–0.79	245	Ref.	288	Ref.	191	Ref.	58	Ref.
	0.79–0.81	258	1.04 (0.85, 1.28)	314	1.09 (0.85, 1.41)	211	1.17 (0.92, 1.48)	56	1.04 (0.70, 1.55)
	0.81–0.83	229	0.93 (0.78, 1.10)	295	0.99 (0.78, 1.27)	189	1.04 (0.85, 1.28)	54	0.98 (0.68, 1.41)
	0.83–0.87	286	1.05 (0.87, 1.27)	371	1.11 (0.89, 1.39)	199	1.01 (0.81, 1.25)	51	0.88 (0.61, 1.26)
Max NDVI (500 m)	0.66–0.76	262	Ref.	307	Ref.	216	Ref.	69	Ref.
	0.76–0.78	230	0.96 (0.77, 1.19)	300	1.06 (0.82, 1.36)	174	0.83 (0.64, 1.07)	45	0.76 (0.51, 1.12)
	0.78–0.81	248	0.99 (0.82, 1.20)	314	1.08 (0.87, 1.34)	181	0.88 (0.72, 1.09)	46	0.73 (0.48, 1.10)
	0.81–0.86	278	1.04 (0.86, 1.27)	347	1.09 (0.84, 1.42)	219	0.94 (0.77, 1.16)	59	0.85 (0.61, 1.19)
Mean NDVI (1000 m)	0.25–0.37	262	Ref.	342	Ref.	208	Ref.	57	Ref.
	0.38–0.44	248	1.00 (0.81, 1.24)	286	0.86 (0.67, 1.10)	189	0.98 (0.78, 1.24)	48	0.99 (0.67, 1.45)
	0.44–0.50	263	1.06 (0.87, 1.30)	316	0.96 (0.75, 1.23)	215	1.05 (0.84, 1.32)	63	1.14 (0.78, 1.66)
	0.50–0.67	245	0.97 (0.81, 1.18)	324	0.92 (0.72, 1.18)	178	0.93 (0.72, 1.19)	51	1.02 (0.69, 1.51)
Mean NDVI (500 m)	0.23–0.36	249	Ref.	320	Ref.	198	Ref.	59	Ref.
	0.36–0.41	275	1.05 (0.85, 1.28)	312	0.91 (0.71, 1.18)	222	1.06 (0.84, 1.33)	53	0.81 (0.56, 1.18)
	0.41–0.48	241	1.02 (0.84, 1.26)	300	0.99 (0.80, 1.22)	181	0.97 (0.79, 1.18)	55	1.05 (0.72, 1.51)
	0.48–0.68	253	1.02 (0.85, 1.22)	336	1.01 (0.80, 1.26)	189	1.02 (0.81, 1.30)	52	0.94 (0.64, 1.39)

*Note.* Linkage data of the 2016–2017 OSDUHS and 2016 environmental data from CANUE. Abbreviations: NDVI, Normalized Difference Vegetation Index; OSDUHS, Ontario Student Drug Use and Health Survey; CANUE, Canadian Urban Environmental Health Research Consortium; aOR, Adjusted odds ratio; 95% CI, 95% confidence interval. ^a^ All models are adjusted for age, sex, ethno-racial background, self-reported socio-economic status, and season.

## References

[1] Woodward L.J., Fergusson D.M. (2001). Life course outcomes of young people with anxiety disorders in adolescence. J. Am. Acad. Child Adolesc. Psychiatry.

[2] Suldo S., Thalji A., Ferron J. (2011). Longitudinal academic outcomes predicted by early adolescents’ subjective well-being, psychopathology, and mental health status yielded from a dual factor model. J. Posit. Psychol..

[3] Government of Canada (2006). The Human Face of Mental Health and Mental Illness in Canada.

[4] Boak A., Hamilton H.A., Adlaf E.M., Henderson J.L., Mann R.E. (2017). The Mental Health and Well-Being of Ontario Students, 1991–2017: Detailed Findings from the Ontario Student Drug Use and Health Survey (OSDUHS) (CAMH Research Document Series No. 47).

[5] (2013). Institute for Health Metrics and Evaluation Global Burden of Diseases, Injuries, and Risk Factors Study. http://ihmeuw.org/4r1u.

[6] James P., Banay R.F., Hart J.E., Laden F. (2015). A Review of the Health Benefits of Greenness. Curr. Epidemiol. Rep..

[7] Triguero-Mas M., Donaire-Gonzalez D., Seto E., Valentín A., Martínez D., Smith G., Hurst G., Carrasco-Turigas G., Masterson D., van den Berg M. (2017). Natural outdoor environments and mental health: Stress as a possible mechanism. Environ. Res..

[8] Dadvand P., Bartoll X., Basagaña X., Dalmau-Bueno A., Martinez D., Ambros A., Cirach M., Triguero-Mas M., Gascon M., Borrell C. (2016). Green spaces and General Health: Roles of mental health status, social support, and physical activity. Environ. Int..

[9] McCormick R. (2017). Does Access to Green Space Impact the Mental Well-being of Children: A Systematic Review. J. Pediatr. Nurs..

[10] Tillmann S., Clark A.F., Gilliland J.A. (2018). Children and Nature: Linking Accessibility of Natural Environments and Children’s Health-Related Quality of Life. Int. J. Environ. Res. Public Health.

[11] Villeneuve P.J., Ysseldyk R.L., Root A., Ambrose S., DiMuzio J., Kumar N., Shehata M., Xi M., Seed E., Li X. (2018). Comparing the Normalized Difference Vegetation Index with the Google Street View Measure of Vegetation to Assess Associations between Greenness, Walkability, Recreational Physical Activity, and Health in Ottawa, Canada. Int. J. Environ. Res. Public Health.

[12] Akpinar A. (2016). How is high school greenness related to students’ restoration and health?. Urban For. Urban Green..

[13] Li D., Sullivan W.C. (2016). Impact of views to school landscapes on recovery from stress and mental fatigue. Landsc. Urban Plan..

[14] Ribeiro A.I., Tavares C., Guttentag A., Barros H. (2019). Association between neighbourhood green space and biological markers in school-aged children. Findings from the Generation XXI birth cohort. Environ. Int..

[15] Kessler R.C., Barker P.R., Colpe L.J., Epstein J.F., Gfroerer J.C., Hiripi E., Howes M.J., Normand S.-L.T., Manderscheid R.W., Walters E.E. (2003). Screening for serious mental illness in the general population. Arch. Gen. Psychiatry.

[16] Peiper N., Clayton R., Wilson R., Illback R. (2015). The performance of the K6 Scale in a large school sample. Psychol. Assess..

[17] Peiper N., Lee A., Lindsay S., Drashner N., Wing J. (2016). The performance of the K6 scale in a large school sample: A follow-up study evaluating measurement invariance on the Idaho Youth Prevention Survey. Psychol. Assess..

[18] Mawani F.N., Gilmour H. (2010). Validation of self-rated mental health. Health Rep..

[19] Substance Abuse and Mental Health Services Administration (2018). 2019 National Survey on Drug Use and Health (NSDUH) Final Screener Specifications for Programming.

[20] Statistics Canada (2009). National Longitudinal Survey of Children and Youth—Cycle 8 Survey Questionnaire.

[21] Rosu A., Chen D. (2016). An improved approach for geocoding Canadian postal code–based data in health-related studies. Can. Geogr..

[22] Kriegler F.J., Malila W.A., Nalepka R.F., Richardson W. Preprocessing Transformations and Their Effects on Multispectral Recognition. Proceedings of the Sixth International Symposium on Remote Sensing of Environment.

[23] Gorelick N., Hancher M., Dixon M., Ilyushchenko S., Thau D., Moore R. (2017). Google Earth Engine: Planetary-scale geospatial analysis for everyone. Remote Sens. Environ..

[24] DMTI Spatial Inc. (2015). CanMap Postal Code Suite v2015.3 [Computer file].

[25] United States Geological Survey Landsat 8 Greenest-Pixel TOA Reflectance Composite, 2013 to 2015 [Data file].

[26] United States Geological Survey Landsat 8 TOA Reflectance (Orthorectified), 2013 to 2017 [Data file].

[27] U.S. Geological Survey Land Processes Distributed Active Archive Center (LP DAAC), MOD13Q1 Vegetation Indices 16-Day L3 Global 250m data [Data file].

[28] Canadian Urban Environmental Health Research Consortium (2018). CANUE Metadata NDVI MODIS.

[29] Pampalon R., Hamel D., Gamache P., Philibert M.D., Raymond G., Simpson A. (2012). An area-based material and social deprivation index for public health in Québec and Canada. Can. J. Public Health Rev. Can. Sante Publique.

[30] Canadian Urban Environmental Health Research Consortium (2019). CANUE Metadata Material and Social Deprivation Index (MSDI).

[31] Gamache P., Hamel D., Pampalon R. (2017). The Material and Social Deprivation Index: A Summary.

[32] Ross N., Wasfi R., Hermann T., Gleckner W. (2018). Canadian Active Living Environments Database (Can-ALE) User Manual & Technical Document.

[33] DMTI Spatial Inc. (2016). CanMap Postal Code Suite v2016.3 [Computer file].

[34] Canadian Urban Environmental Health Research Consortium (2018). CANUE Metadata Canadian Active Living Environments.

[35] Tremblay M.S., Warburton D.E.R., Janssen I., Paterson D.H., Latimer A.E., Rhodes R.E., Kho M.E., Hicks A., LeBlanc A.G., Zehr L. (2011). New Canadian Physical Activity Guidelines. Appl. Physiol. Nutr. Metab..

[36] Akpinar A., Barbosa-Leiker C., Brooks K.R. (2016). Does green space matter? Exploring relationships between green space type and health indicators. Urban For. Urban Green..

[37] Becker D.A., Browning M.H.E.M., Kuo M., Van Den Eeden S.K. (2019). Is green land cover associated with less health care spending? Promising findings from county-level Medicare spending in the continental United States. Urban For. Urban Green..

[38] Francis J., Wood L.J., Knuiman M., Giles-Corti B. (2012). Quality or quantity? Exploring the relationship between Public Open Space attributes and mental health in Perth, Western Australia. Soc. Sci. Med..

[39] Markevych I., Schoierer J., Hartig T., Chudnovsky A., Hystad P., Dzhambov A.M., de Vries S., Triguero-Mas M., Brauer M., Nieuwenhuijsen M.J. (2017). Exploring pathways linking greenspace to health: Theoretical and methodological guidance. Environ. Res..

[40] Houlden V., Weich S., de Albuquerque J.P., Jarvis S., Rees K. (2018). The relationship between greenspace and the mental wellbeing of adults: A systematic review. PLoS ONE.

[41] Rae-grant N., Thomas B.H., Offord D.R., Boyle M.H. (1989). Risk, Protective Factors, and the Prevalence of Behavioral and Emotional Disorders in Children and Adolescents. J. Am. Acad. Child Adolesc. Psychiatry.

